# Supplementary material for: Serious adverse events reported with benzimidazole derivatives: A disproportionality analysis from the World Health Organization’s pharmacovigilance database
Source: PLoS Negl Trop Dis. 2024 Nov 6;18(11):e0012634. doi: 10.1371/journal.pntd.0012634 (PMC11573212; doi:10.1371/journal.pntd.0012634)
Supplement: S1 Material — (DOCX) [file pntd.0012634.s001.docx]

**Serious adverse events reported with benzimidazole derivatives: a disproportionality analysis from the World Health Organization's pharmacovigilance database**

Pamella Modingam^1^, Jean-Luc Faillie^2,3^, Jérémy T. Campillo^1^

1 TransVIHMI, Université de Montpellier, INSERM Unité 1175, Institut de Recherche pour le Développement (IRD), Montpellier, France

2 Pharmacovigilance Regional Center, Department of Medical Pharmacology and Toxicology, CHU Montpellier, Montpellier, France

3 Desbrest Institute of Epidemiology and Public Health, Inserm, Univ Montpellier, Montpellier, France

**Supplementary materials**

**Supplementary Table 1**. Adverse events of interest and corresponding MedDRA terms

**Supplementary Material 1**. Review of the case reports in the literature

**Supplementary Table 2**. Reported MedDRA System Organ Class (SOC) according to anthelmintic drugs type

Corresponding author:

Jeremy T. Campillo

Address: 911 avenue Agropolis, BP 64501, 34394 Montpellier Cedex 5

Email: jeremy.campillo@ird.fr

Phone: +33 4 67 41 61 52

**Supplementary Text 1. Review of case reports in the literature**

To identify new adverse drug reactions (ADRs) associated with benzimidazole derivatives exposure, we conducted a comprehensive literature search using Medline (via PubMed) and Google Scholar. Our search strategy employed keywords combining the names of individual benzimidazole derivatives with terms such as "adverse reaction," "adverse event," and "safety."

The initial search yielded 351 unique documents after removing duplicates. We screened these based on title and abstract, applying the following exclusion criteria:

- Animal studies (n=10)
- In vitro studies (n=10)
- Reviews or meta-analyses (n=36)
- Studies not including benzimidazoles (n=35)
- Studies with irrelevant outcomes (n=154)

This initial screening resulted in 107 potentially relevant articles. We then evaluated 48 full-text articles, further excluding:

- Articles without available full text (n=37)
- Articles with irrelevant outcomes upon closer inspection (n=22)

Finally, we focused on case reports, excluding clinical trials (n=26). This process resulted in 22 relevant case reports for our analysis.

Throughout this process, we critically assessed each article to ensure its relevance to our research question, focusing on new or unusual ADRs associated with benzimidazole derivatives.

Table A:

| **Authors** | **Year** | **Sex** | **Age** | **Drug** | **Dose** | **Adverse event** | **Time of onset** |  |
| --- | --- | --- | --- | --- | --- | --- | --- | --- |
| Bilgic et al. [1] | 2017 | F | 47 | ALB | 400 mg (unique dose) | Vomiting and anorexia | 4 days |  |
|  |  |  |  |  |  |  |  |  |
| Boceanu et al. [2] | 2015 | M | 15 | ALB | 400 mg (unique dose) | Acute liver failure | 14 days |  |
| Xing et al. [3] | 2018 | M | 77 | ALB | MD | Severe pancytopenia | 15 days |  |
| Çaliskan et al. [4] | 2023 | F | 12 | ALB | 400 mg/day | Pruritic erythematous plaques | 5 days |  |
| Choi et al. [5] | 2008 | M | 47 | ALB | 400 mg (unique dose) | Fever, chills, myalgia, nausea, vomiting, and skin rash | 2 days |  |
| Dragutinovic et al. [6] | 2022 | F | 8 | ALB | 400 mg/day | Nausea, Vomiting, Fever | 3 days |  |
|  |  |  |  | MEB | MD | Abdominal pain, nausea, dark urine, elevation of transaminases and cholestasis | 3 days |  |
| Gonzalez-Mendiola et al. [7] | 2007 | F | 52 | MEB | 100 mg (unique dose) | Itching, skin eruptions | 2 hours |  |
| Johnson-Reagan et Bahna [8] | 2003 | F | 7 | THIA | 375 mg/day | Generalized skin rash | 7 days |  |
|  |  | M | 5 |  |  |  | 10 days |  |
|  |  | F | 4 |  |  |  | 21 days |  |
| Marin-Zuluaga et al. [9] | 2013 | F | 25 | ALB | MD | Progressive jaundice, chest pain, fever, icteric sclera, vomiting, weakness, dark urine | 2 weeks |  |
| Mikic et al. [10] | 2009 | F | 27 | ALB | 800 mg/day | Elevation of serum transaminase | 2 months |  |
| Nandi et al. [11] | 2013 | M | 5 | ALB | 400 mg (unique dose) | Fever, vomiting, anorexia, yellowing of eyes and urine | 3 days |  |
| Negi et al. [12] | 2021 | F | 5 | ALB | MD | Yellowing of eyes | 5 days |  |
|  |  | F | 7 |  |  | Yellowing of eyes, vomiting | 4 days |  |
| Pedrosa et al. [13] | 2005 | M | 9 | FLU | MD | Anaphylaxis | 5 days |  |
|  |  |  |  |  |  |  |  |  |
| Riggan et al. [14] | 2020 | M | 53 | ALB | 113.6 g (unique dose) | Alopecia, skin rash, pancytopenia, elevation of transaminases | 3 weeks |  |
| Rios et Restrepo [15] | 2013 | M | 47 | ALB | 600 mg/day | Icteric syndrome, hepatomegaly, dark urine, mucosal jaundice | 5 months |  |
| Metais et al. [16] | 2021 | M | 47 | ALB | 400 mg/day | Asthenia, anorexia and myalgia | 6 days |  |
| Asenov et al. [17] | 2019 | F | 29 | ALB | 10 mg/kg/day | Elevation of transaminases | MD |  |
|  |  | F | 50 | ALB | MD | Elevation of transaminases | MD |  |
|  |  | M | 47 | ALB | MD | Elevation of transaminases | MD |  |
| Nandi et Sarkar [18] | 2013 | M | 5 | ALB | Unique dose | Fever, anorexia, vomiting, yellowish discoloration of eyes and urine | 2-3 weeks |  |
| Koca et Akcam [19] | 2015 | F | 6 | ALB | 15 mg/kg/day | Abdominal pain | 2 months |  |
| Turan et Metin [20] | 2020 | F | 39 | ALB | 1200 mg/day | Alopecia | 2 weeks |  |
|  |  | F | 9 | ALB | 400 mg/day | Alopecia | 2 weeks |  |
| Volpicelli et al. [21] | 2020 | M | 25 | ALB | 200 mg/day | Encephalopathy | 21 days |  |
| Fernandez et al. [22] | 1996 | F | 71 | ALB | 800 mg/day | Pancytopenia | 15 days |  |

MD, missing data; ALB, albendazole; MEB, mebendazole; THIA, thiabendazole; FLU, flubendazole

Age in years

1. Bilgic Y, Yilmaz C, Cagin YF, Atayan Y, Karadag N, Harputluoglu MMM. Albendazole induced recurrent acute toxic hepatitis: a Case report. Acta Gastroenterol Belg. 2017 Apr-Jun;80(2):309-311. PMID: 29560698.

2. Boceanu E, Banateanu M, Tanasescu S, Tamasan I, Marosin F, Pop L. Recurrent acute hepatitis induced by albendazol - Case Report. | Jurnalul Pediatrului 2015, Vol 18, p83

3. Xing F, Ye H, Yang J, Chan JF-W, Seto W-K, Pai PM-C, et al. Fatal pancytopenia due to albendazole treatment for strongyloidiasis. IDCases. 2018;12: 112–116. doi:10.1016/j.idcr.2018.04.002

4. Çalişkan N, Coskun R, Bologur H, Yildırım G, Güngör H, Bosnali O, et al. Treatment of hydatid cyst with albendazole desensitization: A pediatric case report. Indian J Pharmacol. 2023;55: 408–409. doi:10.4103/ijp.ijp_301_23

5. Choi GY, Yang HW, Cho SH, Kang DW, Go H, Lee WC, et al. Acute drug-induced hepatitis caused by albendazole. J Korean Med Sci. 2008;23: 903. doi:10.3346/jkms.2008.23.5.903

6. Dragutinović N, Barać A, Stevanović G, Đorđić I, Paglietti B, Micić J, et al. Acute hepatitis in a paediatric patient: immune-mediated drug-induced liver injury or albendazole-induced autoimmune hepatitis? J Infect Dev Ctries. 2022;16: 1660–1663. doi:10.3855/jidc.16594

7. Gonzalez-Mendiola R, Martínez Borque N, Palomeque Rodríguez T, Torrecillas Toro M, Martínez Bohigas D. Type I allergic reaction to benzimidazole antihelmintics. Allergy. 2007;62: 713–714. doi:10.1111/j.1398-9995.2007.01356.x

8. Johnson-Reagan L, Bahna SL. Severe drug rashes in three siblings simultaneously. Allergy. 2003;58: 445–447. doi:10.1034/j.1398-9995.2003.00120.x

9. Marin Zuluaga JI, Marin Castro AE, Perez Cadavid JC, Restrepo Gutierrez JC. Albendazole-induced granulomatous hepatitis: a case report. J Med Case Reports. 2013;7: 201. doi:10.1186/1752-1947-7-201

10. Mikić D, Jevtić M, Arsić-Komljenović G, Ristanović E, Stanković N, Sjenicić G, et al. [Impossibility of the treatment of inoperable liver multicystic echinococcosis due to adverse reactions to antihelminitics]. Vojnosanit Pregl. 2009;66: 833–839. doi:10.2298/vsp0910833m

11. Nandi M, Sarkar S, Karaagaç AT, Yildirim AI, Sharma J, Mantan M, et al. Correspondence. Indian Pediatr. 2013;50: 1064–1068. doi:10.1007/s13312-013-0285-8

12. Negi B, Thakur P, Sharma Y, Jamwal V, Shekhar C. Drug induced recurrent hepatitis caused by albendazole: Report of two cases. Trop Gastroenterol. 2021;41: 211–213. doi:10.7869/tg.613

13. Pedrosa C, Costa H, Oliveira G, Romariz J, Praça F. Anaphylaxis to povidone in a child. Pediatr Allergy Immunol Off Publ Eur Soc Pediatr Allergy Immunol. 2005;16: 361–362. doi:10.1111/j.1399-3038.2005.00272.x

14. Riggan MAA, Perreault G, Wen A, Raco V, Vassallo S, Gerona R, et al. Case report: Analytically confirmed severe albenzadole overdose presenting with alopecia and pancytopenia. Am J Trop Med Hyg. 2020;102: 177–179. doi:10.4269/ajtmh.19-0198

15. Ríos D, Restrepo JC. Albendazole-induced liver injury: a case report. Colomb Medica. 2013; 118–120. doi:10.25100/cm.v44i2.1021

16. Métais A, Michalak S, Rousseau A. Albendazole-related *Loa Loa* encephalopathy. IDCases. 2021;23: e01033. doi:10.1016/j.idcr.2020.e01033

17. Asenov Y, Akin M, Ibiş C, Tekant Y, Özden I. Observed or predicted albendazole hepatotoxicity as an indication for a resection procedure in hepatic hydatid disease - a Short series of cases. Chir Buchar Rom 1990. 2019;114: 522–527. doi:10.21614/chirurgia.114.4.524

18. Nandi M, Sarkar S. Albendazole-induced recurrent hepatitis. Indian Pediatr. 2013;50: 1064.

19. Koca T, Akcam M. Albendazole-induced autoimmune hepatitis. Indian Pediatr. 2015;52: 78–79.

20. Turan Ç, Metin N. Albendazole-induced anagen effluvium: a brief literature review and our own experience. Acta Dermatovenerol Alp Pannonica Adriat. 2020;29: 161–163.

21. Volpicelli L, De Angelis M, Morano A, Biliotti E, Franchi C, Gabrielli S, et al. Encephalopathy in a patient with loiasis treated with albendazole: A case report. Parasitol Int. 2020;75: 102006. doi:10.1016/j.parint.2019.102006

22. Fernández FJ, Rodríguez-Vidigal FF, Ledesma V, Cabanillas Y, Vagace JM. Aplastic anemia during treatment with albendazole. Am J Hematol. 1996;53: 53–54. doi:10.1002/1096-8652(199609)53:1<53::aid-ajh2830530104>3.0.co;2-q
