# Supplementary material for: Serious adverse events reported with benzimidazole derivatives: A disproportionality analysis from the World Health Organization’s pharmacovigilance database
Source: PLoS Negl Trop Dis. 2024 Nov 6;18(11):e0012634. doi: 10.1371/journal.pntd.0012634 (PMC11573212; doi:10.1371/journal.pntd.0012634)
Supplement: S1 Table — (DOCX) [file pntd.0012634.s002.docx]

**Serious adverse events reported with benzimidazole derivatives: a disproportionality analysis from the World Health Organization's pharmacovigilance database**

Pamella Modingam^1^, Jean-Luc Faillie^2,3^, Jérémy T. Campillo^1^

1 TransVIHMI, Université de Montpellier, INSERM Unité 1175, Institut de Recherche pour le Développement (IRD), Montpellier, France

2 Pharmacovigilance Regional Center, Department of Medical Pharmacology and Toxicology, CHU Montpellier, Montpellier, France

3 Desbrest Institute of Epidemiology and Public Health, Inserm, Univ Montpellier, Montpellier, France

**Supplementary materials**

**Supplementary Table 1**. Adverse events of interest and corresponding MedDRA terms

**Supplementary Material 1**. Review of the case reports in the literature

**Supplementary Table 2**. Reported MedDRA System Organ Class (SOC) according to anthelmintic drugs type

Corresponding author:

Jeremy T. Campillo

Address: 911 avenue Agropolis, BP 64501, 34394 Montpellier Cedex 5

Email: jeremy.campillo@ird.fr

Phone: +33 4 67 41 61 52

**Supplementary Table 1. Adverse events of interest and corresponding MedDRA terms**

| **Adverse reaction of interest** | **MedDRA Terms** |
| --- | --- |
| Serious hepatic disorders | *HLT Hepatocellular damage and hepatitis NEC*  *HLT Hepatic failure and associated disorders*  *HLT Cholestasis and jaundice*  *HLT Hepatic fibrosis and cirrhosis*  *PT Alanine aminotransferase increased*  *PT Aspartate aminotransferase increased*  *PT AST/ALT ratio abnormal*  *PT Bilirubin conjugated increased*  *PT Blood bilirubin increased*  *PT Gamma-glutamyl transferase increased*  *PT Hepatic enzyme increased*  *PT Transaminases abnormal*  *PT Transaminases increased* |
| Hepatitis | *HLT Hepatocellular damage and hepatitis NEC*  *HLT Hepatic failure and associated disorders* |
| Cholecystitis and cholelithiasis | *HLT Cholecystitis and cholelithiasis* |
| Serious nausea and vomiting | *HLT Nausea and vomiting symptoms* |
| Serious cutaneous disorders | *SOC Skin and subcutaneous tissue disorders* |
| Urticaria | *HLT Urticarias* |
| Toxidermias | *PT Drug reaction with eosinophilia and systemic symptoms*  *PT Stevens-Johnson syndrome*  *PT Toxic epidermal necrolysis*  *HLT Bullous conditions*  *PT Acute generalized exanthematous pustulosis* |
| Drug reaction with eosinophilia and systemic symptoms | *PT Drug reaction with eosinophilia and systemic symptoms* |
| Stevens-Johnson syndrome | *PT Stevens-Johnson syndrome* |
| Toxic epidermal necrolysis | *PT Toxic epidermal necrolysis* |
| Bullous conditions | *HLT Bullous conditions* |
| Acute generalized exanthematous pustulosis | *PT Acute generalized exanthematous pustulosis* |
| Serious allergic reactions | *HLT Allergic conditions NEC*  *HLT Anaphylactic and anaphylactoid responses*  *HLT Angioedemas* |
| Seizures | *HLT Generalized tonic-clonic seizure*  *HLT Partial simple seizures NEC*  *HLT Seizures and seizure disorders NEC* |
| Leucopenia | *HLT Neutropenias*  *PT Leukopenia*  *PT Neutrophil count decreased*  *PT White blood cell count decreased* |
| Marrow depression and hypoplastic anaemias | *HLT Marrow depression and hypoplastic anaemias* |
| Headaches | *PT Headache* |
| Dizziness | *PT Dizziness* |
