# Supplementary material for: Serious adverse events reported with benzimidazole derivatives: A disproportionality analysis from the World Health Organization’s pharmacovigilance database
Source: PLoS Negl Trop Dis. 2024 Nov 6;18(11):e0012634. doi: 10.1371/journal.pntd.0012634 (PMC11573212; doi:10.1371/journal.pntd.0012634)
Supplement: S2 Table — (DOCX) [file pntd.0012634.s003.docx]

**Serious adverse events reported with benzimidazole derivatives: a disproportionality analysis from the World Health Organization's pharmacovigilance database**

Pamella Modingam^1^, Jean-Luc Faillie^2,3^, Jérémy T. Campillo^1^

1 TransVIHMI, Université de Montpellier, INSERM Unité 1175, Institut de Recherche pour le Développement (IRD), Montpellier, France

2 Pharmacovigilance Regional Center, Department of Medical Pharmacology and Toxicology, CHU Montpellier, Montpellier, France

3 Desbrest Institute of Epidemiology and Public Health, Inserm, Univ Montpellier, Montpellier, France

**Supplementary materials**

**Supplementary Table 1**. Adverse events of interest and corresponding MedDRA terms

**Supplementary Material 1**. Review of the case reports in the literature

**Supplementary Table 2**. Reported MedDRA System Organ Class (SOC) according to anthelmintic drugs type

**Supplementary Table 2. Reported MedDRA System Organ Class (SOC) according to anthelmintic drugs type**

| **MedDRA SOC name** | **Albendazole**  N = 11,212^1^ | **Mebendazole**  N = 6,118^1^ | **Flubendazole**  N = 951^1^ | **Thiabendazole**  N = 1,614^1^ | **Triclabendazole**  N = 92^1^ | **Ivermectin** N = 55,188^1^ | **Praziquantel** N = 5,865^1^ | **Overall** N = 81,040^1^ |
| --- | --- | --- | --- | --- | --- | --- | --- | --- |
| Gastrointestinal disorders | 3,022 (27%) | 1,911 (31%) | 318 (33%) | 360 (22%) | 12 (13%) | 8,553 (15%) | 2,307 (39%) | 16,483 (20%) |
| Skin and subcutaneous tissue disorders | 1,909 (17%) | 1,047 (17%) | 171 (18%) | 244 (15%) | 8 (8.7%) | 12,363 (22%) | 721 (12%) | 16,463 (20%) |
| General disorders and administration site conditions | 1,757 (16%) | 742 (12%) | 114 (12%) | 190 (12%) | 25 (27%) | 9,813 (18%) | 678 (12%) | 13,319 (16%) |
| Nervous system disorders | 1,406 (13%) | 644 (11%) | 66 (6.9%) | 244 (15%) | 5 (5.4%) | 6,204 (11%) | 1,263 (22%) | 9,832 (12%) |
| Injury, poisoning and procedural complications | 329 (2.9%) | 369 (6.0%) | 123 (13%) | 12 (0.7%) | 9 (9.8%) | 5,409 (9.8%) | 48 (0.8%) | 6,299 (7.8%) |
| Eye disorders | 212 (1.9%) | 92 (1.5%) | 13 (1.4%) | 60 (3.7%) | 0 (0%) | 1,581 (2.9%) | 101 (1.7%) | 2,059 (2.5%) |
| Infections and infestations | 235 (2.1%) | 155 (2.5%) | 15 (1.6%) | 52 (3.2%) | 11 (12%) | 1,536 (2.8%) | 40 (0.7%) | 2,044 (2.5%) |
| Musculoskeletal and connective tissue disorders | 226 (2.0%) | 114 (1.9%) | 7 (0.7%) | 20 (1.2%) | 3 (3.3%) | 1,479 (2.7%) | 86 (1.5%) | 1,935 (2.4%) |
| Psychiatric disorders | 192 (1.7%) | 109 (1.8%) | 7 (0.7%) | 82 (5.1%) | 6 (6.5%) | 1,275 (2.3%) | 36 (0.6%) | 1,707 (2.1%) |
| Respiratory, thoracic and mediastinal disorders | 210 (1.9%) | 121 (2.0%) | 24 (2.5%) | 26 (1.6%) | 1 (1.1%) | 1,236 (2.2%) | 83 (1.4%) | 1,701 (2.1%) |
| Investigations | 368 (3.3%) | 96 (1.6%) | 9 (0.9%) | 46 (2.9%) | 5 (5.4%) | 924 (1.7%) | 43 (0.7%) | 1,491 (1.8%) |
| Hepatobiliary disorders | 379 (3.4%) | 82 (1.3%) | 14 (1.5%) | 84 (5.2%) | 2 (2.2%) | 453 (0.8%) | 33 (0.6%) | 1,047 (1.3%) |
| Metabolism and nutrition disorders | 111 (1.0%) | 60 (1.0%) | 6 (0.6%) | 20 (1.2%) | 1 (1.1%) | 759 (1.4%) | 58 (1.0%) | 1,015 (1.3%) |
| Renal and urinary disorders | 74 (0.7%) | 48 (0.8%) | 0 (0%) | 30 (1.9%) | 0 (0%) | 579 (1.0%) | 12 (0.2%) | 743 (0.9%) |
| Blood and lymphatic system disorders | 285 (2.5%) | 45 (0.7%) | 3 (0.3%) | 24 (1.5%) | 0 (0%) | 354 (0.6%) | 23 (0.4%) | 734 (0.9%) |
| Cardiac disorders | 64 (0.6%) | 29 (0.5%) | 2 (0.2%) | 26 (1.6%) | 0 (0%) | 573 (1.0%) | 36 (0.6%) | 730 (0.9%) |
| Vascular disorders | 78 (0.7%) | 40 (0.7%) | 5 (0.5%) | 16 (1.0%) | 1 (1.1%) | 555 (1.0%) | 25 (0.4%) | 720 (0.9%) |
| Immune system disorders | 101 (0.9%) | 92 (1.5%) | 11 (1.2%) | 4 (0.2%) | 1 (1.1%) | 348 (0.6%) | 138 (2.4%) | 695 (0.9%) |
| Ear and labyrinth disorders | 100 (0.9%) | 49 (0.8%) | 10 (1.1%) | 64 (4.0%) | 1 (1.1%) | 297 (0.5%) | 111 (1.9%) | 632 (0.8%) |
| Product issues | 45 (0.4%) | 41 (0.7%) | 3 (0.3%) | 0 (0%) | 0 (0%) | 258 (0.5%) | 5 (<0.1%) | 352 (0.4%) |
| Reproductive system and breast disorders | 37 (0.3%) | 37 (0.6%) | 2 (0.2%) | 2 (0.1%) | 1 (1.1%) | 225 (0.4%) | 8 (0.1%) | 312 (0.4%) |
| Pregnancy, puerperium and perinatal conditions | 15 (0.1%) | 83 (1.4%) | 12 (1.3%) | 0 (0%) | 0 (0%) | 75 (0.1%) | 1 (<0.1%) | 186 (0.2%) |
| Surgical and medical procedures | 25 (0.2%) | 12 (0.2%) | 2 (0.2%) | 0 (0%) | 0 (0%) | 132 (0.2%) | 2 (<0.1%) | 173 (0.2%) |
| Congenital, familial and genetic disorders | 9 (<0.1%) | 83 (1.4%) | 12 (1.3%) | 0 (0%) | 0 (0%) | 12 (<0.1%) | 0 (0%) | 116 (0.1%) |
| Neoplasms benign, malignant and unspecified (incl cysts and polyps) | 2 (<0.1%) | 13 (0.2%) | 1 (0.1%) | 2 (0.1%) | 0 (0%) | 87 (0.2%) | 0 (0%) | 105 (0.1%) |
| Social circumstances | 14 (0.1%) | 4 (<0.1%) | 1 (0.1%) | 2 (0.1%) | 0 (0%) | 72 (0.1%) | 5 (<0.1%) | 98 (0.1%) |
| Endocrine disorders | 7 (<0.1%) | 0 (0%) | 0 (0%) | 4 (0.2%) | 0 (0%) | 36 (<0.1%) | 2 (<0.1%) | 49 (<0.1%) |
| ^1^ N, Total of adverse events reported in each SOC. A same ICSR can contain multiple MedDRA SOC. | | | | | | | | |
