# Supplementary material for: Serious adverse events reported with benzimidazole derivatives: A disproportionality analysis from the World Health Organization’s pharmacovigilance database
Source: PLoS Negl Trop Dis. 2024 Nov 6;18(11):e0012634. doi: 10.1371/journal.pntd.0012634 (PMC11573212; doi:10.1371/journal.pntd.0012634)
Supplement: Attachment — Submitted filename: PNTD-D-24-01059_Comments.docx [file pntd.0012634.s004.docx]

Countries are encouraged to review their safety data and regularly monitor the safety of drugs and vaccines and identify new signals. This article is very relevant since it gives a practical example on the way aggregated safety data can be reviewed to check for new signals.

However, the methods need more clarification to ease reading and reproducibility. In particular, there is a need for more consistency in the pharmacovigilance terminologies.

**General**

The manuscript is about severe adverse drug reactions (L1) associated (L1), related (L147), or linked (L166) to benzimidazole derivatives. However not all ICSRs in VigiBase has causality assessment to confirm relatedness. Even causality assessment per se is not enough to confirm association. Neither disproportionality analysis which is used to “identify disproportionate reporting, defined as a higher-than-expected number of ADR reports compared other reactions recorded in the database” (L117). It is a first step to identify potential quantitative signals that need further assessment for confirmation and action.

Therefore, unless you are confident about causality or relatedness, we would suggest you use less suggestive terms. May be adverse events, adverse events of special interest, reported instead of related, etc.

You will also need to use consistently the term serious and severe. For example, the same events are described as serious in table 3 but severe elsewhere in the manuscript.

**Data source**

L140: You need to provide more details on the way you extracted data from the WHO global database. If you used the VigiLyze tool you need to state it (with precision if you used the full dataset or the de-duplicated dataset) and provide the link. You will also need to provide the filters used to allow for reproducibility.

109: It maybe more relevant to explain that these variables are the ones available in the line-listing extracted from VigiBase or VigiLyze, therefore to be included in multivariate analysis.

**Case definition:**

- Cases were reports of suspected serious adverse drug reactions (ADR) with benzimidazole derivatives (L43),
- Cases were serious ADRs of interest (L135).

You should have one definition of cases.

It is also important to be more consistent and explain if each ADR of interest is a case or if each ICSR with at least 1 ADR of interest is a case, i.e. an ICSR with 2 ADRs of interest (headaches and seizures) is counted once or twice?

**Non-case:**

- Non-cases were reports with other anthelmintic drugs (L44);
- Non-cases were defined as reports of any other suspected ADR than the ADR of interest (L143).

You should have one definition for non-case

I am not sure it is a good practice to include the exposure in the definition of case or non-case.

I would suggest you also include serious in the definition of non-case.

**Comparator:**

L151 Instead of saying “compared to all other selected anthelmintic drugs reported in VigiBase”, isn’t it easier to say compared to PZQ and IVM, chosen as comparators due to their similar indications (L129).

**Statistical analysis**

L50: Calculating the ROR of each suspected ADR of interest lead to a multiplicity of analysis which increases the risk of type 1 error. Please make sure you implemented corrective methods.

**Denominator:**

It is not clear if the denominator is the total number of serious ADRs or the total number of ICSRs for the corresponding drug.

The confusion with denominator raises because there is no consistency in the use of case, report, ICSR, ADR, etc. ICSR is clearly defined in L107 but poorly used in the manuscript. Note that this difference is relevant since one patient may have several ADRs. In Supplementary table 2, the denominator for each drug is the total number of events by MedDRA SOC, not the total number of ICSRs. What is the same for the other tables?

For further clarification, we suggest an additional supplementary table which displays ADR of interest and other ADRs per anthelmintic drugs type, including the denominator for each drug. This new table will also support understanding of table 2.
