# Supplementary material for: Serious adverse events reported with benzimidazole derivatives: A disproportionality analysis from the World Health Organization’s pharmacovigilance database
Source: PLoS Negl Trop Dis. 2024 Nov 6;18(11):e0012634. doi: 10.1371/journal.pntd.0012634 (PMC11573212; doi:10.1371/journal.pntd.0012634)
Supplement: Attachment — Submitted filename: Revision Letter_Plos-PV-Alb jlf+jc.docx [file pntd.0012634.s005.docx]

September the 16^th^, 2024

Eduardo José Lopes-Torres

Academic Editor

*PLOS Neglected Tropical Diseases*

Jong-Yil Chai

Section Editor

*PLOS Neglected Tropical Diseases*

Dear Editors,

We hereby submit the revised version of our manuscript entitled “**Serious adverse drug reactions associated with benzimidazole derivatives: a disproportionality analysis from the World Health Organization's pharmacovigilance database**” (PNTD-D-24-01059). We thank the two reviewers and the executive editors for their positive comments and appreciation of the paper.

We addressed each of the comments with detailed responses. We hope our manuscript will now meet the standards for publication.

Sincerely yours,

Jérémy CAMPILLO

jeremy.campillo@ird.fr

911 avenue Agropolis, 34000, Montpellier, France

*Tel: +33 4 67 41 61 62*

________________________________________________________________________________

**Reviewer #1**.

*Countries are encouraged to review their safety data and regularly monitor the safety of drugs and vaccines and identify new signals. This article is very relevant since it gives a practical example on the way aggregated safety data can be reviewed to check for new signals.*

*However, the methods need more clarification to ease reading and reproducibility. In particular, there is a need for more consistency in the pharmacovigilance terminologies.*

*The manuscript is about severe adverse drug reactions (L1) associated (L1), related (L147), or linked (L166) to benzimidazole derivatives. However not all ICSRs in VigiBase has causality assessment to confirm relatedness. Even causality assessment per se is not enough to confirm association. Neither disproportionality analysis which is used to “identify disproportionate reporting, defined as a higher-than-expected number of ADR reports compared other reactions recorded in the database” (L117). It is a first step to identify potential quantitative signals that need further assessment for confirmation and action. Therefore, unless you are confident about causality or relatedness, we would suggest you use less suggestive terms. May be adverse events, adverse events of special interest, reported instead of related, etc. You will also need to use consistently the term serious and severe. For example, the same events are described as serious in table 3 but severe elsewhere in the manuscript.*

We would like to thank the Reviewer for his/her valuable feedback on our manuscript. We appreciate his/her recognition of the relevance of our work. We have carefully considered the comments and made the proposed modifications to enhance the clarity and reproducibility of our methods. Specifically, we have ensured consistency in the use of pharmacovigilance terminologies throughout the manuscript. Regarding the terminology used to describe “adverse drug reactions”, we have revised the manuscript and the title to use “adverse events” instead of “adverse reactions”, we also revised the manuscript to use “reported” instead of “related” or “associated”. Additionally, we have ensured consistent use of the terms “serious” across the manuscript.

*Data source*

*L140: You need to provide more details on the way you extracted data from the WHO global database. If you used the VigiLyze tool you need to state it (with precision if you used the full dataset or the de-duplicated dataset) and provide the link. You will also need to provide the filters used to allow for reproducibility.*

We did not use the VigiLyze tool for data extraction. Instead, a de-duplicated dataset was specifically provided by the Uppsala Monitoring Centre upon request. As such, we cannot provide a direct link for extraction. The term "deduplicated" was indeed missing, and we have now added it to the corresponding section. Additionally, no filters were applied beyond those specified in the manuscript, which included only serious ICSRs with anthelmintics, as defined by Anatomical Therapeutic Chemical (ATC) codes P02CA, D01AC, P02BX, and P02BA, and covering the period from January 1, 2000, to December 31, 2023.

*109: It maybe more relevant to explain that these variables are the ones available in the line-listing extracted from VigiBase or VigiLyze, therefore to be included in multivariate analysis.*

We thank the Reviewer for his/her suggestion. We have revised the sentence to clarify that the variables included in the multivariate analysis were those available in the line-listing extracted from VigiBase.

*Case definition:*

*Cases were reports of suspected serious adverse drug reactions (ADR) with benzimidazole derivatives (L43),*

*Cases were serious ADRs of interest (L135).*

*You should have one definition of cases.*

We have revised the definition of cases to ensure consistency throughout the manuscript. The updated definition is: “Cases were ICSRs where at least one suspected serious adverse event of interest was reported, while non-cases were ICSRs reporting any adverse events other than the serious adverse events of interest.”

*It is also important to be more consistent and explain if each ADR of interest is a case or if each ICSR with at least 1 ADR of interest is a case, i.e. an ICSR with 2 ADRs of interest (headaches and seizures) is counted once or twice?*

We have clarified in the manuscript that an ICSR is considered a case if it includes at least one AE of interest. Consequently, an ICSR containing multiple AE of interest (e.g., headaches and seizures) is counted once for each AE of interest in the respective analyses. The manuscript now includes the following clarifications:

- "An ICSR is an anonymized report for a single individual who experienced adverse event(s) potentially linked to the use of one or more drugs."

- "An ICSR can contain multiple adverse events"

- "For each analysis, cases were ICSRs where at least one suspected serious adverse event of interest was reported."

*Non-case:*

*Non-cases were reports with other anthelmintic drugs (L44);*

*Non-cases were defined as reports of any other suspected ADR than the ADR of interest (L143).*

*You should have one definition for non-case*

*I am not sure it is a good practice to include the exposure in the definition of case or non-case.*

*I would suggest you also include serious in the definition of non-case.*

We thank the Reviewer for highlighting this issue. It is correct that the definition of "non-case" should not include exposure. As mentioned above, we have revised the definition to clarify that "non-cases were defined as ICSRs reporting any adverse events other than the serious adverse events of interest." This correction has been applied throughout the manuscript.

*Comparator:*

*L151 Instead of saying “compared to all other selected anthelmintic drugs reported in VigiBase”, isn’t it easier to say compared to PZQ and IVM, chosen as comparators due to their similar indications (L129).*

We have revised the text which now reads: “compared to PZQ and IVM.”

*Statistical analysis*

*L50: Calculating the ROR of each suspected ADR of interest lead to a multiplicity of analysis which increases the risk of type 1 error. Please make sure you implemented corrective methods.*

We acknowledge that the multiplicity of analyses increases the risk of Type I errors. Although we did not apply formal corrective methods for multiple comparisons, we focused on 17 specific adverse events selected based on a literature review and preliminary frequency analysis in our dataset. Our consistent findings across various sub-analyses suggest a low likelihood of random statistically significant results. We have added this comment to the discussion section to address this concern.

*Denominator:*

*It is not clear if the denominator is the total number of serious ADRs or the total number of ICSRs for the corresponding drug.*

*The confusion with denominator raises because there is no consistency in the use of case, report, ICSR, ADR, etc. ICSR is clearly defined in L107 but poorly used in the manuscript. Note that this difference is relevant since one patient may have several ADRs. In Supplementary table 2, the denominator for each drug is the total number of events by MedDRA SOC, not the total number of ICSRs. What is the same for the other tables?*

*For further clarification, we suggest an additional supplementary table which displays ADR of interest and other ADRs per anthelmintic drugs type, including the denominator for each drug. This new table will also support understanding of table 2.*

We have revised the manuscript and the tables to address the reviewer concerns. For all analyses, the denominator used is the total number of ICSRs. In Supplementary Table 2, the denominator is the total number of adverse events reported in each SOC. We have ensured consistency in the use of « ICSRs » or « adverse events » throughout the manuscript to avoid any confusion. We hope these modifications make the manuscript clearer and address the reviewer concerns. We believe that Supplementary Table 2 and Table 2 already address the reviewer’s suggestion of including information about the denominator for each drug.

_________________________________________________________________________________

**Reviewer #2**.

*The authors reported the results of a disproportionality analysis using the World Health Organization's pharmacovigilance database regarding benzimidazole derivatives for the first time. The manuscript is well written with several subanalyses.*

*I only had few comments:*

*-	In the Discussion section, the authors stated “severe leukopenia, severe liver disorders, hepatitis, severe hepatic disorders, severe urticaria, severe convulsions/seizures, dizziness, severe nausea and vomiting”, which is misleading as the ADR in the Vigibase are “serious” and not “severe”. Indeed, an ADR could be considered serious in the Vigibase if it is a “medically important event or reaction”, independent of any notion of severity.*

We thank the Reviewer for highlighting this issue. We made the requested modifications ensuring consistent use of the terms “serious” across the manuscript.

*-	The authors could elaborate on possible pathophysiological mechanisms at the origin of the ADR found, even if this not the purpose of a disproportionality analysis.*

We added a paragraph about potential pathophysiological mechanisms in the discussion: “The pathophysiological mechanisms underlying the suspected adverse events following exposure to benzimidazole derivatives remain incompletely understood. For hematological disorders such as bone marrow failure, hypoplastic anemia, leukopenia, and pancytopenia, the primary hypothesis is that this class of drugs can inhibit tubulin polymerization, potentially impairing cell turnover and leading to a depletion of cell lineages. Additionally, benzimidazoles may interfere with folate metabolism, further contributing to hematological toxicity. Additionally, benzimidazoles may interfere with folate metabolism, further contributing to hematological toxicity. Hepatic disorders, which are well-known adverse drug reactions, might be linked to the drug’s metabolites, primarily eliminated via the biliary route. The mechanisms underlying seizures and skin disorders are unclear and need further investigations. One hypothesis is that these effects might involve the direct action of the drug on parasites in the blood (such as loiasis) or in brain (such as taeniasis), provoking allergic and/or neurologic reactions due to parasite death, circulation of their remains, or embolization in brain capillaries”.

*-	The authors should discuss possible protopathic bias, as several ADR found can be symptoms of parasitic infections (liver disorders, seizures, urticaria…)*

We thank the reviewer for this very relevant comment. We have addressed this point by adding the following paragraph in the discussion section: “It is crucial to consider the potential for protopathic bias in interpreting our results. Several adverse effects, such as liver disorders, seizures, or urticaria can also be symptoms of parasitic infections for which benzimidazole derivatives are prescribed. This could lead to misattribution of disease symptoms to the drug, potentially inflating the apparent association with benzimidazole use.”

*-	The authors should provide further details on the literature review: which “expert pharmacovigilants”? which keywords? which search engines?…*

We have provided further details on our literature review process by including comprehensive information in Supplementary Material 2.

*-	Some corrections need to be done: typos (a new signals, as well-tolerated, Table 2 shows describes, according anthelminthic drugs,…) ; inconsistencies (in abstract: aROR sometimes written, sometimes not) ; “other benzimidazole derivatives (i)” in the Discussion section?; legend 1 not explained in Supplementary Table 2; article 22 misquoted ; “of which 5,131 cases” instead of 5,137.*

We thank the reviewer for their careful attention. We have made the requested corrections.__________________________________________________________________________________

**Editors.**

*Concerns were raised about the adequacy of the sample size in relation to the power of the analysis.*

*While the figures and tables are generally clear, there may be room for improvement in their presentation.*

*Given these points, the manuscript is being returned with a recommendation for Major Revision. Please address the reviewers' comments in detail and resubmit your revised manuscript for further consideration.*

Thanks to the reviewers' valuable comments, we have improved the overall quality of the manuscript and tables. Regarding the concern about sample size, we would like to note that calculating a sample size is not applicable for disproportionality analysis, as this type of analysis uses all available data in the pharmacovigilance database to detect signals rather than predefined sample sizes. Nevertheless, we remain available should the editor request any further changes, whether in form or substance.
